# Validation of a parent HPV vaccine misperceptions scale and its association with children’s HPV vaccination status

**DOI:** 10.1016/j.vaccine.2025.127616

**Published:** 2025-08-24

**Authors:** Corinne McDaniels-Davidson, Humberto Parada, Maria Elena Martinez, Lourdes S. Martinez, Jesse N. Nodora, Margaux Stack-Babich, Olivia Keleman, Emily E. Miller, Jennifer K. Felner, David Strong

**Affiliations:** a Division of Health Promotion and Behavioral Science, School of Public Health, San Diego State University, San Diego, CA 92182, United States; b UC San Diego Health Moores Cancer Center, La Jolla, CA 92037, United States; c Department of Radiation Medicine and Applied Sciences, School of Medicine, University of California, San Diego, La Jolla, CA 92093, United States; d Division of Epidemiology, School of Public Health, San Diego State University, San Diego, CA 92182, United States; e Herbert Wertheim School of Public Health and Human Longevity Science, University of California, San Diego, La Jolla, CA 92093, United States; f School of Communication, San Diego State University, San Diego, CA 92182, United States

**Keywords:** HPV vaccine, Scale, Vaccine hesitancy, Patient decision making, Vaccine hesitancy scale

## Abstract

Human papillomavirus (HPV) infection is the cause of nearly all cervical and anal cancers and the majority of vaginal, oropharyngeal, vulvar, and penile cancers in the United States (US). A safe and effective vaccine for the most common cancer-causing HPV types has been available in the US since 2006; however, uptake among age-eligible children remains below that of vaccines administered at similar ages. Hesitancy fueled by misperceptions about the HPV vaccine may contribute to this gap. We assessed HPV vaccine misperceptions using a 12-item Likert scale in a population health assessment in a large county in California. We validated the scale using exploratory factor analysis and exploratory graph analysis. We further assessed concurrent validity by examining parent report of youth vaccination through weighted logistic regression. The HPV Vaccine Misperceptions scale had high internal consistency (Cronbach’s alpha = 0.94) and a strong primary dimension. Further, the 12-item scale sum score was associated with increased odds (Odds Ratio_per_ SD = 2.09; 95 % Confidence Interval = 1.26–3.45) of age-eligible children being unvaccinated for HPV in multivariable-adjusted logistic regression models. This Parent HPV Vaccine Misperceptions Scale can be used to identify parent barriers to vaccination for tailored health education to increase uptake of the HPV vaccine in age-eligible children.

## 1. Background

Human Papillomavirus (HPV) is a common virus transmitted through skin-to-skin contact [1], though only about 40 of the more than 100 identified HPV types infect mucous membranes [2]. While most of these HPV types are benign, 15 are considered high-risk types because they cause nearly all cervical and anal cancers and most vaginal, vulvar, oropharyngeal, and penile cancers [3,4].

Safe and effective vaccines for certain HPV types have been approved for use in the United States (US) since 2006 [5]. The current vaccine formulation (Gardasil 9) prevents the seven most common high risk HPV types and the two most common low risk HPV types (responsible for the vast majority of US genital warts cases) [6]. A two-dose vaccine series is available for 9–14 year olds; 15–26 year olds require a 3-dose series [7]. Vaccine series initiation and completion in US adolescents have lagged other high-income countries [8] due in part to vaccine hesitancy and common misperceptions [9], with steady recent gains set back during the COVID-19 pandemic [10].

Vaccine hesitancy is multifaceted and may be due to incorrect knowledge, beliefs, and attitudes about the vaccine that are promoted by exposure to HPV vaccine misinformation [11]. HPV vaccine misinformation is false information that is unintentionally or purposefully shared [12], but parents may not realize the information is false. When parents adopt HPV vaccine misinformation, they develop “false beliefs” that form the basis for HPV vaccine misperceptions. For example, parents may form beliefs that the vaccine is age-inappropriate [13], have concerns about vaccine safety [14,15], or associate the vaccine with stigmatizing beliefs about sexuality [16,17] based on HPV vaccine misinformation they encountered in their information environment. Collectively the adoption of false information into false beliefs about HPV vaccination provides the basis for misperceptions about the HPV vaccine.

Several instruments have been developed to assess elements of parent HPV vaccine knowledge and hesitancy [18–22], though few have been validated against actual assessments of HPV vaccine administration. While the 8-item Vaccination Confidence Scale was associated with provider report of HPV vaccination, the items are broadly applicable to vaccines and so may fail to pinpoint specific issues that parents have with the HPV vaccine [18]. The Adapted Vaccine Hesitancy Scale-HPV is specific to the HPV vaccine, but concurrent validation was limited to association with a parent question about whether concerns ever stopped them from getting their child the HPV vaccine [19]. It is critical that the public health and medical fields are equipped with assessments of hesitancy or misperceptions that are associated with vaccine receipt. This knowledge provides practitioners with the ability to identify and discuss concerns with parents and patients, and ideally lead to increased vaccination. This understanding also facilitates tailored public health campaigns for HPV vaccination.

Given the limitations of previously developed HPV vaccination scales, we sought to validate a new HPV Vaccine Misperception Scale and to determine whether the scale sum score was associated with parent report of age-eligible children’s HPV vaccine status using a weighted population-based assessment.

## 2. Methods

### 2.1. Participants

We conducted a cross-sectional population health assessment in spring 2019: the San Diego County Assessment to Reach Equal Health Status (SD CARES) [23]. The goal of SD CARES was to assess cancer-related knowledge, attitudes, and behaviors in a representative sample of residents living in San Diego County, California. San Diego County is the southwestern-most county in the US and is the fifth largest US county by population. A stratified random sample of 5000 county households (including 4000 randomly selected county households and an additional 1000 households randomly selected from ZIP codes along the US-Mexico border) received a study packet through First Class Mail. Study packets included both English and Spanish materials, including invitation letters with informed consent, 12-page survey booklets, a business reply envelope, and a $2 bill pre-incentive. The exterior of the envelope was stamped with “Gift Enclosed.” Instructions stated that for households with more than one adult, the adult with the next upcoming birthday should complete the survey. A reminder letter was sent a few months later and included instructions to complete and return the original survey, request a new survey, or complete the survey online if preferred (an option added for the reminder letter). The assessment yielded a 14.4 % response rate (*n* = 720), with 685 paper surveys returned by business reply and 35 completed online. Data were weighted by ICF International (Fairfax, VA) using a three-dimensional raking approach with age, gender, and race/ethnicity as post-stratification variables to ensure representativeness to the San Diego County population [24].

#### Measures.

**Fig. 1.**
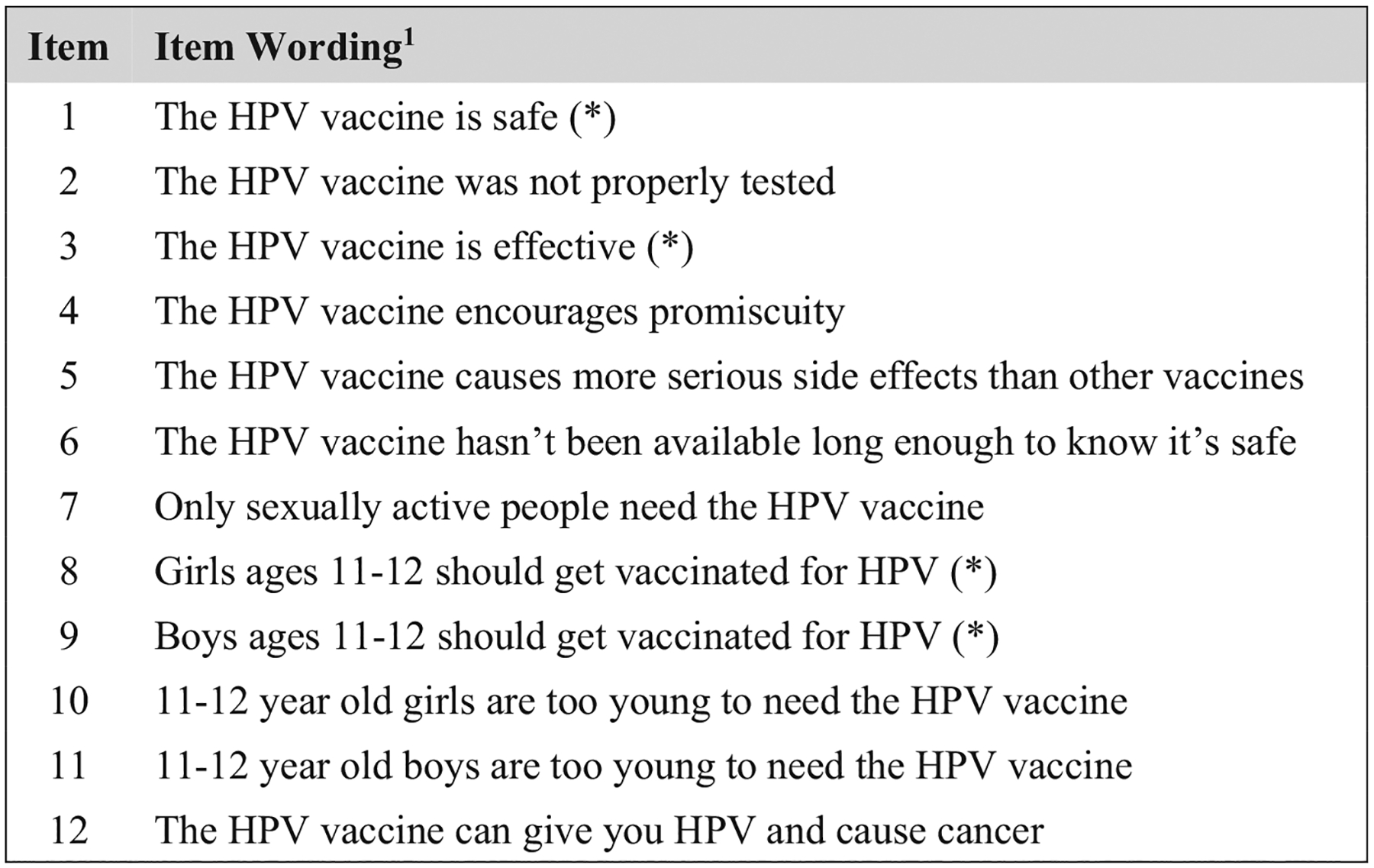
HPV Vaccine Misperception Scale items.

HPV vaccine misperceptions were measured using 12 Likert scale items shown in Fig. 1. Item responses were coded as 0 = strongly disagree, 1 = disagree, 2 = agree, 3 = strongly agree, with the exception of four reverse coded items (marked with asterisks). Sum scores could range from 0 to 36 with higher values indicating greater agreement with HPV vaccine misperceptions. Items were adopted from those used in a previous National Cancer Institute (NCI)-funded population health assessment [25] fielded by an NCI-designated comprehensive cancer center (unpublished). Item domains were designed to capture a single common dimension underlying misperceptions of safety, effectiveness, knowledge of eligibility, and concerns about the HPV vaccine promoting promiscuity or causing cancer.

[1] Response options: strongly disagree, disagree, agree, strongly agree

#### * Indicates item is reverse coded for scoring.

Age-eligible children’s vaccination status was assessed through a series of parent/guardian-reported questions. Those that responded affirmatively to “*Do you have a daughter 9–26 years old?*” were then asked, “*Has your daughter 9–26 years old been vaccinated for HPV? If you have more than one daughter 9–26 years old, please answer about your youngest daughter in the age range*.” The questions were repeated for a son 9–26 years. A vaccination status variable was then created only for those with age-eligible children. Those with only a daughter who was vaccinated, only a son who was vaccinated, or with both a daughter and son who were both vaccinated were classified as vaccinated. Those with mixed vaccination or who reported no vaccination were classified as not vaccinated.

Household income, parent race and ethnicity, and parent education level were self-reported by the parent. Categorical household income was dichotomized into <$75,000 per year and ≥ $75,000 per year. Parent education level was dichotomized into less than a college degree and college graduate or higher. Race and ethnicity were categorized as Hispanic/Latino, non-Hispanic Asian/Pacific Islander, non-Hispanic Black, and non-Hispanic white.

### 2.2. Statistical analysis

Descriptive statistics of scale items and the overall scale sum score were assessed through item response frequencies and total score means and standard deviations. Construct validity and support for a single summed misperceptions scale score was assessed using exploratory factor analysis and exploratory graph analysis (EGA) of pairwise associations within the network of items that were adjusted for other items in the model [26]. The strength of a proposed single primary factor relative to other extracted factors was determined using an eigenvalue cutoff of 1.0, visual examination of scree plots, the cumulative percent of variance explained by the first relative to remaining factors, and parallel analysis comparing observed to simulated solutions [27]. EFA and EGA included comparison of models with a single primary underlying factor relative to a model with separate correlated factors using a hierarchical bifactor arrangement of items [26]. Relative fit to single factor and bifactor models that isolate a primary dimension and allow residual item variation to organize within smaller specific subfactors was assessed using change in Akaike Information Criteria (AIC), Bayesian Information Criterion (BIC) factor models and Total Entropy Fit Index for EGA network models [28]. Item analyses used full information maximum likelihood (FIML) estimation implemented by ‘mirt’ [29], which includes all response patterns, including cases with some missing item responses [30]. Cases with all missing scale items (*n* = 109) were excluded from the analysis.

**Fig. 2.**
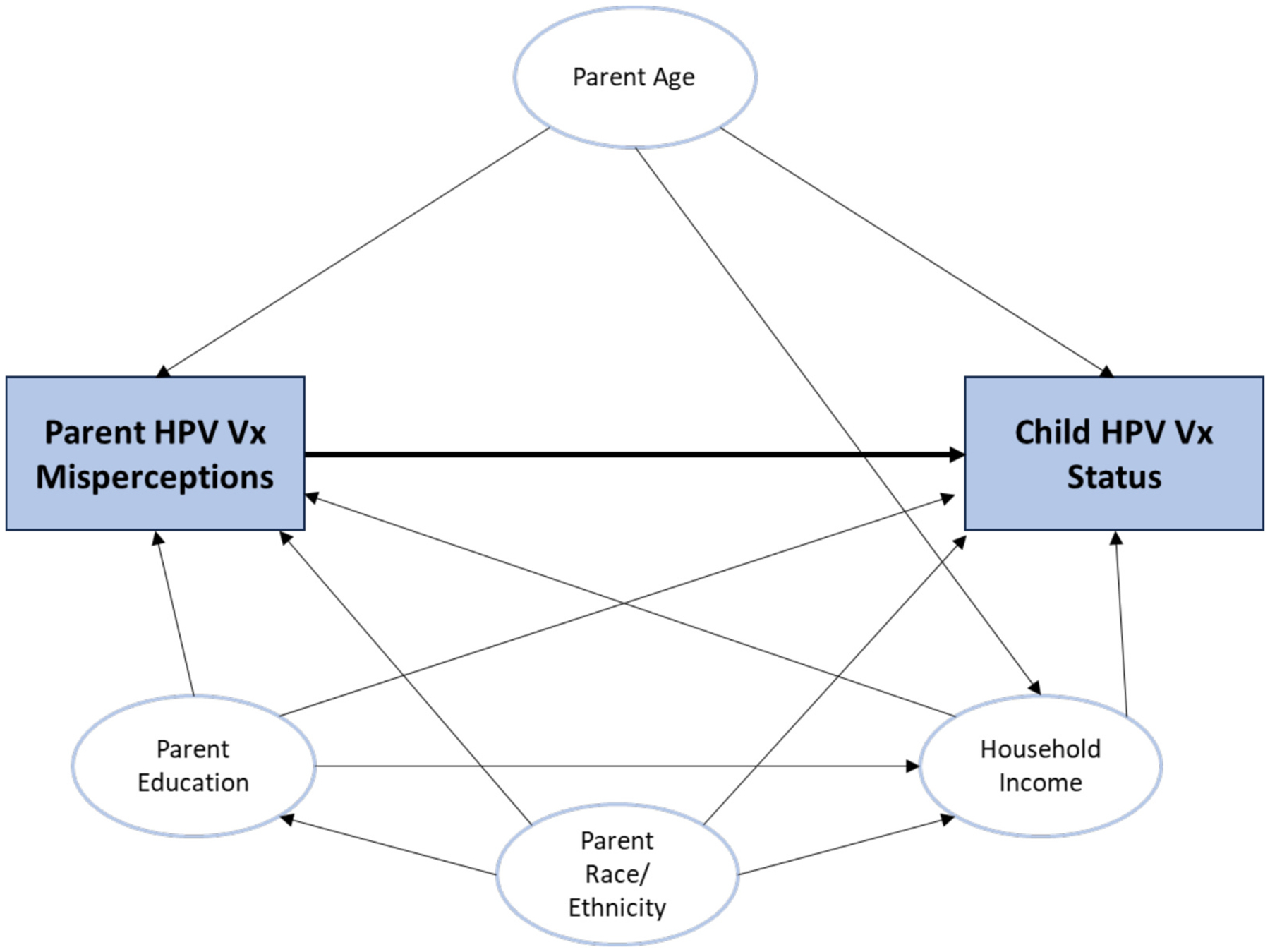
Directed Acyclic Graph (DAG) depicting hypothesized causal pathway between parent HPV Vaccine Misperceptions sum score and their age-eligible child (ren)’s HPV vaccine status.

Concurrent validity of the HPV Vaccine Misperception Scale was evaluated by determining the extent to which the scale sum score of parents with age-eligible (9–26 years) children was associated with the HPV vaccination status of their age-eligible children. Weighted means and frequencies were used to explore univariate variables and bivariate associations by whether parents reported that their age-eligible child (ren) received the HPV vaccine. To test the hypothesis that higher parent HPV Vaccine Misperception sum scores were associated with their child (ren)’s HPV vaccination status, survey-weighted crude and multivariable-adjusted logistic regression models were built to estimate odds ratios (ORs) and 95 % confidence intervals (CIs). Multivariable models adjusted for demographic variables hypothesized to be associated with both parent misperceptions and children’s vaccination status (Fig. 2) [31], including parent education level, household income, and race/ethnicity. All analyses were completed with IBM SPSS Statistics v28 (descriptive statistics), R statistical software (EFA and EGA) [32], and SAS v9.4 (SAS Institute Inc., Cary, NC; weighted logistic regression).

## 3. Results

The 12-item HPV Vaccine Misperception Scale within SD CARES was completed by 611 community members. Item response frequencies indicating agreement with each statement differed across the twelve items (Table 1). Missingness varied from 5.7 % (*n* = 35; Item 1) to 12.1 % (*n* = 74; Items 2 and 5).

### 3.1. Strength of a single misperceptions dimension

The Kaiser-Meyer-Olkin (KMO) Measure of Sampling Adequacy was 0.85 for the community sample, suggesting that the use of factor analysis was appropriate [33]. Initial factor extraction on the 12-item set yielded initial eigenvalues of 5.60, 0.74, 0.55, and 0.21. Parallel analysis suggested these four eigenvalues were significantly larger than those extracted from simulated data which were 0.52, 0.18, 0.13, 0.10 respectively. The first eigenvalue was 7.57 times larger than the second and was the only value exceeding criteria of >1.0. EGA also suggested four communities of items (group A: items 2,4–7 & 12; group B: items 1,3; group C: items 8,9; group D: 10,11) were best organized using a single primary dimension using a hierarchical bifactor model (TEFI = −16.73) rather than a model with four separate correlated communities (TEFI = 4.72). Using factor analysis, a bifactor model arranging items as suggested by EGA resulted in improved model fit (delta AIC = −1122; delta BIC = −1069). Loadings on the primary factor were consistent across single and bifactor models (see Table 2) and support a single primary scale.

Item loadings were consistently strong and ranged from 0.56 to 0.93. Items 4 and 7 which incorrectly tie the HPV vaccine to sexual activity and promiscuity had the weakest loadings and lowest communalities (h2). Items that had the strongest loadings and largest communalities were focused on the age recommendations for the vaccine and centered around the perceived lack of safety and downstream effects of the vaccine. Estimates of reliability suggest a strong primary dimension and omega hierarchical [34] of 0.80 relative to the total omega of 0.97. Cronbach’s coefficient alpha [35] was 0.94 suggesting good internal consistency of scores from this scale.

### 3.2. Concurrent validity

With the scale evaluation and internal validation complete, validation of the tool against vaccination status was conducted in the weighted sample of parents with HPV vaccine age-eligible children (n_weighted_ = 516,563). Table 3 presents weighted descriptive characteristics of the target population of parents with children age-eligible for the HPV vaccine by whether the parent reported that their age-eligible child was vaccinated for HPV. The overall parent mean age was 48.4 ± 9.1 years. The mean HPV Vaccination Sum Score was higher among those that reported not vaccinating their children for HPV (15.3 ± 7.5) compared to those with vaccinated children (10.6 ± 6.2).

The parent HPV Vaccine Misperceptions Sum Score was associated with their child(ren)’s HPV vaccination status in unadjusted and fully adjusted models. In unadjusted survey-weighted logistic regression models, the odds that a parent’s age-eligible child(ren) were not vaccinated against HPV doubled for every standard deviation increase in the HPV Vaccine Misperception Sum Score (Table 4). The measure of association remained unchanged in models adjusted for the minimal sufficient set as suggested by our DAG (Fig. 2; parent education level, household income, and race/ethnicity).

## 4. Discussion

The HPV Vaccine Misperceptions Scale is a reliable and valid 12-item tool with a single misperception dimension. Further, parent sum score was associated with the HPV vaccination status of their age-eligible child(ren) in this weighted population-based assessment in Southern California. For each standard deviation increase in the parent scale sum score, the odds of their child(ren) not being vaccinated against HPV doubled. This association remained consistent in fully adjusted models, demonstrating concurrent validity.

While several parent vaccine scales have been proposed [18–20,22,36], few demonstrate validity against the outcome of HPV vaccination. The Vaccine Confidence Scale is an 8-item tool that assesses overall vaccine confidence [18]. This scale has been associated with provider reported HPV vaccination, however, the items themselves are non-specific to HPV vaccination, limiting its public health and health communication utility. The Adapted Vaccine Hesitancy Scale-HPV is a 9-item tool assessing HPV vaccine confidence and perceived risks. While it is associated with parent reports of concerns about the vaccine stopping them from getting their child vaccinated, it has not been linked explicitly to child HPV vaccine receipt [19]. The Modified Parent Attitudes about Childhood Vaccines (PACV) scale is an 18-item tool assessing overall vaccine hesitancy that has not been assessed for concurrent validity with HPV vaccine receipt [20]. Last, the Vaccine Conspiracy Beliefs Scale is a 7-item assessment of belief in conspiracies that was validated in a Canadian sample with parent willingness to vaccinate their sons [22]. Among those that have demonstrated concurrent validity, none have done so in a large racially and ethnically diverse population. Our HPV Vaccine Misperceptions Scale was validated in both community and parent samples, with strikingly similar results. This suggests that community perceptions can be an analog to parent perceptions, as we previously suggested [23].

The study had several strengths. Our large sample size was sufficient for validation of the HPV Vaccine Misperceptions Scale. The population-based sample is another study strength, as is the sample weighting for the assessment of concurrent validity, alleviating non-response bias. In addition, the sample was diverse in terms of racial and ethnic make-up, improving the generalizability of results. However, the present findings should be interpreted with study limitations in mind. The relatively low response rate could have resulted in non-response bias, though survey weights did yield close similarities between our weighted population and 2017 census estimates on measures such as household income. As we adopted item wording from a previous instrument, we did not conduct qualitative assessment of the items among the population. Additionally, the cross-sectional self-report surveys were completed by a single parent, meaning the sociodemographic information represents only one parent and only their HPV vaccine misperceptions were assessed, though parent dyads often make vaccination decisions together. It should also be noted that for those parents with multiple age-eligible children, vaccination information was only gathered about the youngest son (if there were multiple sons) and the youngest daughter (in the case of multiple daughters). Similarly, the sample size limited the ability to explore HPV vaccine misperceptions where there was discordance in son/daughter vaccination; these were collapsed into “not vaccinated”. Previous work suggests parent-reported HPV vaccination status is subject to false negatives (reporting no vaccination when children have been vaccinated), particularly among non-white and low SES parents [37,38]. However, false negative HPV vaccination reporting would bias our results toward the null hypothesis, bolstering the findings of an association between misperceptions and child vaccination status. In addition, the present study examined only personal beliefs; we were unable to assess physician trust or physician recommendation, which is an important predictor of HPV vaccination status [39]. Those choosing to implement this scale in the future may consider modifying the age-related questions to focus on age 9 rather than 11–12-year-olds, as recommendations shift to earlier vaccination [40].

Our HPV Vaccine Misperceptions Scale is a valid measure, demonstrating concurrent validity with self-reported vaccination status of age-eligible children. Replication in other geographies and with other populations is important for external validity. This simple tool could be used for population level public health and personal health education. It identifies concerns that can be addressed by health education or health care professionals. Correcting misperceptions is critical to achieving nationwide HPV vaccination goals and to continue toward the elimination of HPV-related cancers.

## Figures and Tables

**Table 1:** Response distribution of HPV Vaccine Misperception Scale items among community members (*n* = 611).

Item	Strongly disagree	Disagree	Agree	Strongly agree	Missing
1*	24 (4 %)	73 (13 %)	349 (61 %)	130 (23 %)	35
2	108 (20 %)	323 (60 %)	83 (15 %)	23 (4.3 %)	74
3*	16 (3 %)	70 (13 %)	358 (66 %)	96 (18 %)	71
4	300 (53 %)	191 (33 %)	54 (9.5 %)	26 (4.6 %)	40
5	129 (24 %)	315 (59 %)	69 (13 %)	24 (4.5 %)	74
6	98 (18 %)	245 (44 %)	166 (30 %)	45 (8.1 %)	57
7	244 (44 %)	231 (42 %)	69 (12 %)	12 (2.2 %)	55
8*	49 (9 %)	99 (18 %)	249 (44 %)	166 (29 %)	48
9*	51 (9 %)	107 (19 %)	166 (29 %)	157 (28 %)	49
10	181 (32 %)	242 (43 %)	96 (17 %)	38 (6.8 %)	54
11	174 (31 %)	242 (44 %)	96 (17 %)	41 (7.4 %)	58
12	207 (38 %)	278 (52 %)	38 (7.1 %)	16 (3.0 %)	72

* Indicates item reverse coded for scoring.

**Table 2:** Factor loadings of HPV Vaccine Misperceptions Scale items in community members (*n* = 611).

	Single Factor		Hierarchical Bifactor		
Item	F1	h2	G	h2	Item Group
1	0.87	0.75	0.86	0.90	A
2	0.73	0.53	0.71	0.61	B
3	0.86	0.74	0.83	1.00	A
4	0.56	0.31	0.50	0.52	B
5	0.79	0.62	0.75	0.85	B
6	0.73	0.54	0.70	0.69	B
7	0.56	0.32	0.52	0.39	B
8	0.90	0.80	0.83	0.97	C
9	0.89	0.80	0.82	1.00	C
10	0.93	0.86	0.82	1.00	D
11	0.93	0.86	0.82	0.97	D
12	0.70	0.50	0.66	0.58	B

Note: F1: factor loading for item from single factor model; G: factor loading on primary single factor with adjustment for the presence of 4 specific grouping factors using the bifactor model. h2: item communality reflecting percentage of item variance in common with other modeled items.

**Table 3:** Weighted^1^ characteristics of the target population of parents with children age-eligible for the HPV vaccine by whether their age-eligible child(ren) received the HPV vaccine (*n* = 516,563).

	Age-eligible child vaccination status					
Characteristics, mean (SD) or No. (%)	Not Vaccinated (*n* = 231,231)		Vaccinated (*n* = 285,332)		All (n = 516,563)	
**Parent age, years**	46.8	(9.8)	49.6	(8.3)	48.4	(9.1)
**Parent sex**						
Male	120.798	(53.2)	73,040	(26.2)	193,838	(38.3)
Female	106,491	(46.9)	205,890	(73.8)	312,381	(61.7)
**Parent race/ethnicity**						
Hispanic	107,996	(46.7)	124,068	(43.5)	232,064	(44.9)
Non-Hispanic Asian/Pacific Islander	33,703	(14.6)	33,703	(21.1)	93,976	(18.2)
Non-Hispanic Black	7327	(3.2)	7327	(4.4)	20,009	(3.9)
Non-Hispanic white	82,205	(35.5)	82,205	(31.0)	170,513	(33.0)
**Annual household income**						
<$75,000	109,387	(47.3)	83,580	(29.3)	192,967	(37.4)
≥$75,000	121,844	(52.7)	201,752	(70.7)	323,596	(62.6)
**Parent education**						
Less than college degree	118,806	(51.4)	126,865	(44.5)	245,670	(47.6)
College degree or higher	112,425	(48.6)	158,467	(55.5)	270,892	(52.4)
**HPV Vaccine Misperceptions Sum Score**	15.3	(7.5)	10.6	(6.2)	12.7	(7.2)

1 Weighted frequencies are rounded to the nearest whole number SD: standard deviation.

**Table 4:** Odds of age-eligible children being unvaccinated for HPV by HPV Vaccine Misperception Sum Score (*n* = 516,563).

Modeled measure of perceived benefits or risks	Unadjusted model			Fully-Adjusted model^a^		
	OR	95 % CI	*P* value	OR	95 % CI	*p* value
HPV Vaccine Misperceptions Sum Score, per SD	2.01	1.30–3.13	0.002	2.09	1.26–3.45	0.004

a Adjusted for minimal sufficient set: parent education level, household income, and race/ethnicity. SD: standard deviation, OR: odds ratio, CI: confidence interval.

## Data Availability

Data will be made available on request.
